# The Effect of Oleoylethanolamide (OEA) Add-On Treatment on Inflammatory, Oxidative Stress, Lipid, and Biochemical Parameters in the Acute Ischemic Stroke Patients: Randomized Double-Blind Placebo-Controlled Study

**DOI:** 10.1155/2022/5721167

**Published:** 2022-09-08

**Authors:** Mohammadmahdi Sabahi, Sara Ami Ahmadi, Azin Kazemi, Maryam Mehrpooya, Mojtaba Khazaei, Akram Ranjbar, Ashkan Mowla

**Affiliations:** ^1^Neurosurgery Research Group (NRG), Student Research Committee, Hamadan University of Medical Sciences, Hamadan, Iran; ^2^Behavioral Disorders and Substances Abuse Research Center, Hamadan University of Medical Sciences, Hamadan, Iran; ^3^Department of Clinical Pharmacy, School of Pharmacy, Hamadan University of Medical Sciences, Hamadan, Iran; ^4^Department of Neurology, School of Medicine, Hamadan University of Medical Sciences, Hamadan, Iran; ^5^Department of Pharmacology and Toxicology, School of Pharmacy, Hamadan University of Medical Sciences, Hamadan, Iran; ^6^Nutrition Health Research Center, Hamadan University of Medical Sciences, Hamadan, Iran; ^7^Department of Neurological Surgery, Keck School of Medicine, University of Southern California (USC), Los Angeles, CA, USA

## Abstract

**Methods:**

Sixty patients with a mean age of 68.60 ± 2.10 comprising 29 females (48.33%), who were admitted to an academic tertiary care facility within the first 12 hours poststroke symptoms onset or last known well (LKW), in case symptom onset time is not clear, were included in this study. AIS was confirmed based on a noncontrast head CT scan and also neurological symptoms. Patients were randomly and blindly assigned to OEA of 300 mg/day (*n* = 20) or 600 mg/day (*n* = 20) or placebo (*n* = 20) in addition to the standard AIS treatment for three days. A blood sample was drawn at 12 hours from symptoms onset or LKW as the baseline followed by the second blood sample at 72 hours post symptoms onset or LKW. Blood samples were assessed for inflammatory and biochemical parameters, oxidative stress (OS) biomarkers, and lipid profile.

**Results:**

Compared to the baseline, there is a significant reduction in the urea, creatinine, triglyceride, high-density lipoprotein, cholesterol, alanine transaminase, total antioxidant capacity, malondialdehyde (MDA), total thiol groups (TTG), interleukin-6 (IL-6), and C-reactive protein levels on the follow-up blood testing in the OEA (300 mg/day) group. In patients receiving OEA (600 mg/day) treatment, there was only a significant reduction in the MDA level comparing baseline with follow-up blood testing. Also, the between-group analysis revealed a statistically significant difference between patients receiving OEA (300 mg/day) and placebo in terms of IL-6 and TTG level reduction when comparing them between baseline and follow-up blood testing.

**Conclusion:**

OEA in moderate dosage, 300 mg/day, add-on to the standard stroke treatment improves short-term inflammatory, OS, lipid, and biochemical parameters in patients with AIS. This effect might lead to a better long-term neurological prognosis.

## 1. Introduction

The prevalence and mortality of noncommunicable diseases have surpassed infectious diseases globally. Neurological disorders such as stroke, Alzheimer's, and Parkinson's are associated with high mortality and morbidity, and thus far, there is no cure for them. These disorders have revealed common pathological features such as inflammation, oxidative stress (OS) production, abnormal protein accumulation, disruption of normal calcium homeostasis, and apoptosis. Among these, stroke is one of the leading causes of death worldwide and a major cause of disability in adults [1–3].

Stroke is a neurological disorder that is divided into ischemic and hemorrhagic types [4, 5]. In the ischemic type, the hypoxia of the brain tissue occurs with the cessation of blood flow to the brain tissue and leads to the destruction of neurons and glial cells [2, 3, 6, 7]. The sequence of the events responding to ischemia is known as the ischemic cascade, including glutamate release, calcium influx, OS, inflammation, and ultimately, apoptosis, which leads to irreversible neuronal death [8, 9].

Oleoylethanolamide (OEA) is a member of the N-acylethanolamine (NAE) family, which are endogenously bioactive lipids and are formed from amidation of membrane phospholipids. This fatty acid is mostly found in sources such as olives and sesame [10, 11]. Pure olive oil counteracts cell death by reducing lipid peroxidation (LPO), brain prostaglandin E2, and nitric oxide production, while increasing glutathione concentrations. The family of NAEs is involved in a wide range of body processes such as inflammation, nerve protection, acute stress, pain relief, anxiety, hypotension, sleep, and energy balance [11]. Based on previous findings, using olive oil fatty acid supplements reduces damage and protects the brain tissue during an ischemic stroke [3]. Peroxisome proliferator-activated receptor alpha (PPAR-*α*) and cannabinoid receptors (CBR) have critical roles in regulating inflammations [12]. The activation of the CBR1 can protect neural tissues against ischemic injury or tissue reperfusion injury by reducing OS, releasing lactate dehydrogenase, and activating caspase-3 in vitro [13]. The members of the NAE family have been identified as the natural ligands of the CBR. In addition to the CBR, OEA can bind to PPAR-*α*, and consequently, exert its anti-inflammatory effects [12], since PPAR-*α* acts as a negative regulator of the inflammatory response through direct binding to the p65–nuclear factor (NF)-*κ*B, which subsequently antagonizes NF-*κ*B transcription factor pathways [14, 15].

This study sought to determine the effect of OEA supplementation on the biomarkers of OS, inflammatory parameters, lipid profile, and renal and hepatic parameters in patients with acute ischemic stroke.

## 2. Materials and Methods

### 2.1. Trial Setting and Design

This single-center three-day randomized, placebo-controlled, double-blind, parallel-group trial was conducted in Farshchian (Sina) Hospital, an affiliate of the Hamadan University of Medical Sciences, Hamadan, Iran. All patients were screened and enrolled between April 21^st^, 2020 and July 22^nd^, 2020.

### 2.2. Standard Protocol Approvals, Registrations, and Patient Consents

The trial was registered in the Iranian Registry of Clinical Trials (Identifier: IRCT20130501013194N4) and approved by the Ethics Committee of the Hamadan University of Medical Sciences with the ethical code of IR.UMSHA.REC.1398.720-722. Before participation in the study, all participants and/or their next of kin signed a written informed consent form in compliance with the Declaration of Helsinki.

### 2.3. Patients

**Figure 1:**
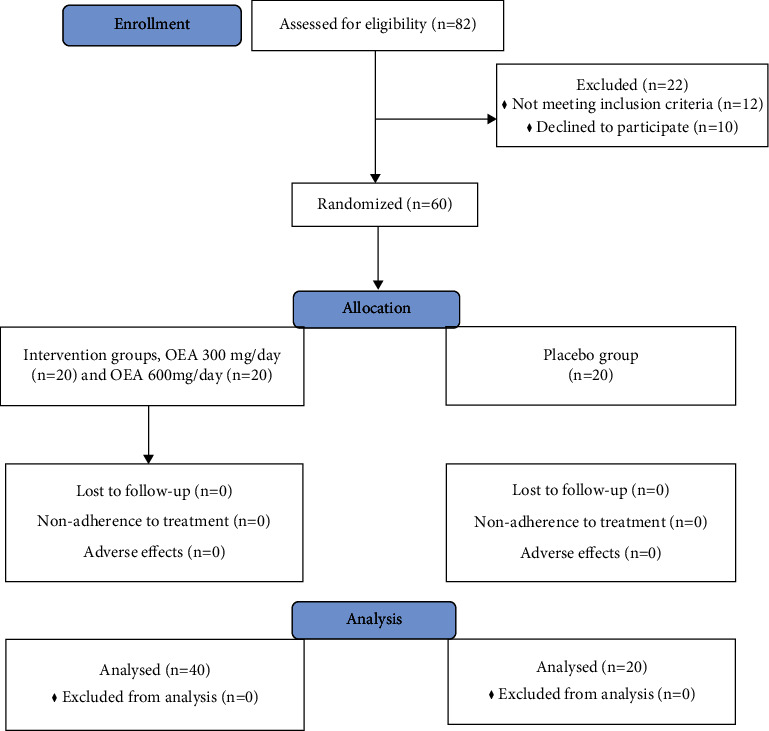
The flow diagram of the study.

In general, 82 patients were assessed for their eligibility; among whom, 12 and 10 patients did not meet the inclusion criteria or declined to participate, respectively. As such, 60 patients aged 30-93 years who met the inclusion and exclusion criteria were consented to participate in the study (Figure 1). No patients lost to follow-up since they were all admitted in the hospital for at least three days after the onset of their neurological symptoms or last known well (LKW), in case stroke onset time was unclear. The National Institute of the Health Stroke Scale (NIHSS) score and the modified Rankin Scale (mRS) were used to measure the neurological deficits and functional outcome poststroke, respectively. The NIHSS is a scoring system which offers a quantitative assessment neurological deficit caused by stroke. This 15-item scale measures the deficits caused by acute stroke on several domains including awareness, language, eye movements, motor force, ataxia, and sensorium. Each item is graded on a three- to five-point scale leading to scores between 0 and 42, where 0 indicates no deficit and 42 translates to death [16]. Furthermore, the mRS is a functional outcome measure, which ranges from 0 to 6, where 0 defines as no neurological symptoms and 6 indicates death [17].

The patients were included if they were reaching the emergency department within 12 hours from the neurological symptom onset or LKW in case the onset time was unclear, had no evidence of intracranial hemorrhage on their initial noncontrast head CT, and had NIHSS score ≥2 and <20 [18]. Exclusion criteria is as follows: patients aged <18, had a temperate ≥38.0°C and/or a white blood cell count ≥16,000 cells/mm^3^, given we did this study during the COVID pandemic, history or suspicion of active malignancy, inflammatory vasculopathy, systemic inflammatory disease, connective tissue disease, hypercoagulable state, and chronic renal failure, as well as patients who had a glomerular filtration rate <30, hepatic failure and/or cirrhosis, gastrointestinal bleeding, uncontrolled diabetes mellitus, chronic respiratory diseases, hematologic illnesses, and seizure at presentation. Also, use of medications/supplements, having either anti-inflammatory or anti-OS effect, prior sensitivity to compounds, alcohol and drug abuse, and a vegetative state were the other exclusion criteria.

These 60 patients underwent quick neurological assessment upon admission, as per standard practice [19, 20]. Patients meeting both inclusion and exclusion criteria were divided into two categories. Patients who were eligible for acute reperfusion therapy including intravenous and/or intra-arterial thrombolytic therapy and those who were not eligible for acute treatment. In both categories, in addition to OEA or placebo, patients' underlying diseases such as diabetes mellitus, hypertension, and dyslipidemia were addressed as per standard of care. They also receive antiplatelets or anticoagulants when appropriate as per standard of care. Patients were not allowed to receive any additional treatment in the course of the trial.

The baseline blood sample was drawn at 12 hours after LKW, testing OS, and lipid, along with renal and hepatic parameters during the acute phase. The follow-up sample was drawn at 72 hours after LKW.

Patients were randomly divided into three groups using Minitab software, patients who received OEA 300 mg/day, 600 mg/day, or placebo capsules with respect to concealment, respectively. All three groups received the treatment for three consecutive days. Each individual received a computer code, and the code was disclosed during the treatment period. The allocation was hidden behind a series of sequentially numbered, opaque, and sealed envelopes. A study coordinator opened the envelope after the recruitment of each patient. The patients were randomly assigned and assessed by two physicians. The statistician, the patients, the referring physician, and the physician evaluating the patients and providing the trial drugs, were all blinded to the allocation. The treatment codes were revealed at the end of the study and after closing the database. All three groups were adjusted for age and gender, extent and severity of ischemic stroke using the NIHSS score, baseline mRS, acute infarct volumes on noncontrast head CT, door to needle time in case intravenous thrombolytic therapy was administered, whether acute reperfusion therapy was performed, body mass index, history of smoking, and history of hypertension. Furthermore, groups were adjusted for history of hyperlipidemia, coronary artery disease, diabetes mellitus, deep vein thrombosis, prior stroke, systolic and diastolic blood pressure, and home medication prior to admission (Table 1). Frequency matching methods were used for this purpose.

### 2.4. Chemicals

The applied chemicals in this investigation included 5,5'-Dithiobis-(2-nitrobenzoic acid) (DTNB), 2,4,6-tripyridyl-S-triazine (TPTZ), Thiobarbituric acid (TBA), Nitro blue tetrazolium (NBT), and sodium phosphate buffer that were obtained from the Sigma-Aldrich (St. Louis, MO, USA). OEA supplements were provided by Karen Pharma and Food Supplement Co.

### 2.5. Biochemical Analysis

Phlebotomy was employed to collect ten milliliters of venous blood into chilled ethylenediaminetetraacetic acid-containing tubes, which were then centrifuged for plasma separation, aliquoted in 1.5 ml vials, snap-frozen, and kept at −80°C until further analyses. After the treatment period, interleukin- (IL-) 6 levels were measured in the blood samples of the subjects by enzyme-linked immunosorbent assay, and C-reactive protein (CRP) levels were measured by turbidometry. Portion of the blood sample was sent to the laboratory to measure the lipid profile including triglycerides (TG), total cholesterol, and high-density lipoprotein (HDL) in addition to serum hematological parameters comprising urea, creatinine, aspartate transaminase (AST), and alanine transaminase (ALT). On the other portion of the blood sample, the following parameters were measured: LPO level by assessing malondialdehyde (MDA), capacity of the serum antioxidants which is called total antioxidant capacity (TAC), the level of total thiol groups (TTG), and the activity of the superoxide dismutase (SOD) enzyme by a spectrophotometer using the TBA reagent at 532 nm, the TPTZ reagent at 593 nm, the DTNB reagent at 412 nm, and the NBT reagent at 560 nm wavelength, respectively.

### 2.6. Statistical Analysis

Descriptive analysis and comparison of differences between demographics in each group were performed by SPSS 16 software. The mean, standard deviation, frequency, and percentage were used to represent the data. The Kolmogorov-Smirnov test was applied to determine if the distribution was normal. The Chi-squared test and Fisher's exact test were employed for comparing qualitative variables between the groups. In addition, the independent *t*-test and Mann–Whitney *U* test were used to compare quantitative variables. Furthermore, intragroup differences between baseline and follow-up parameters were examined using the Wilcoxon signed-rank test. Finally, the between-group difference analysis was conducted through the Kruskal-Wallis test, followed by a post hoc test, and *P* values less than 0.05 were considered statistically significant.

### 2.7. Data Availability Policy

Any competent investigator can request anonymized data from the corresponding author for replicating techniques and findings.

## 3. Results

Thirty-one men (51.66%) and 29 women (48.33%) participated in this study, and their mean age was 68.60 ± 2.10 (range 30-93). Demographic and clinical characteristics of the study participants including the cardiovascular risk factors, acute ischemic stroke event characteristics, medications prior to admission, and the underlying mechanism for stroke are summarized in Table 1. No significant differences were observed between the baseline characteristics of these three groups. Our primary outcome was to evaluate the effect of OEA on inflammatory and OS parameters. The secondary outcome was to investigate the effect of OEA on the lipid profile, as well as renal and hepatic parameters.

### 3.1. Renal Parameters

As shown in Table 2, although the between-group analysis revealed no statistically significant difference in the blood urea and creatinine levels, there was a statistically significant decline in the level of urea in patients receiving 300 mg/day OEA. Overall, OEA 300 mg/day reduced both urea and creatinine levels and might act as a renoprotective factor in acute ischemic stroke patients.

### 3.2. Lipid Profile

As demonstrated in Table 2, a statistically significant reduction in total cholesterol and TG and a rise in HDL levels were seen in patients receiving OEA 300 mg/day, while changes in other groups were not statistically significant in either between-group or within-group analysis.

### 3.3. Hepatic Parameters

As shown in Table 2, although between-group analysis revealed no statistically significant difference regarding ALT and AST levels, there was a statistically significant decline in the level of ALT in patients receiving OEA 300 mg/day.

### 3.4. OS Parameters

The levels of OS parameters are shown in Table 2. Although SOD levels have no significant difference between the groups, within-group analysis indicated that consumption of OEA could significantly alter TAC, MDA only in the 300 mg/day group, and TTG in both 300 mg/day and 600 mg/day groups. Also, between-group analysis indicated statistically significant differences in TTG, and the results of post hoc test revealed that this difference was noticeable between patients receiving OEA 300 mg/day and a placebo (*P* = 0.047).

### 3.5. Inflammatory Profile

The levels of inflammatory parameters are displayed in Table 2. In within-group analysis, patients who received moderate dose of OEA, 300 mg/day, showed statistically significant decrease in IL-6 and CRP levels. Between-group analysis demonstrated statistically significant differences in IL-6, and based on the results of post hoc test, this difference was observed between patients receiving OEA, 300 mg/day, and placebo (*P* = 0.037).

### 3.6. Complications

Table 3 presents the occurrence of adverse effects. Nausea, vomiting, dyspepsia, and headache were the most frequently reported adverse effects among the participants regardless of their treatment group. There were no statistically significant differences among different groups regarding the reported side effects.

## 4. Discussion

OS and inflammation are considered as an important pathophysiological mechanism in acute ischemic stroke. OS is a consequence of the failure in the equilibrium between the endogenous reactive oxygen species (ROS) production and their cleansing by endogenous antioxidant defense systems [21]. The rapid increase in ROS production immediately after acute ischemic stroke quickly overwhelms the antioxidant capacity of the brain tissue and causes further tissue damage. ROS can damage cell macromolecules, resulting in autophagy, apoptosis, and necrosis. Furthermore, the rapid restoration of the blood flow after reperfusion increases the level of oxygen delivery to the tissue, leading to a new wave of ROS production and thus further tissue damage [22].

Inflammation is also one of the main pathological mechanisms of acute ischemic stroke and may cause OS [23]. Various cytokines such as tumor necrosis factor (TNF), IL-1, and IL-6 are known to regulate the tissue damage in stroke models, and therefore, play a crucial role in poststroke therapies. The effect of these cytokines on the development and progression of infarction in human and animal models depends on various factors, including their availability in the penumbra of the stroke area at the onset of early symptoms [24].

Diet and lifestyle are known to play important roles in preventing noncommunicable diseases. High-fat diets may cause metabolic changes, and obesity can induce chronic inflammation. Diet-induced obesity and related metabolic disorders such as hyperlipidemia are also considered risk factors for cardiovascular diseases and stroke [25].

A Mediterranean diet, which has widely known to be beneficial on health, is a diet that is characterized by the presence of useful bioactive compounds such as monounsaturated fatty acids (MUFAs) and polyunsaturated fatty acids or polyphenols [26].

OEA is derived from the unsaturated fatty acid oleic acid, which is part of MUFA. This fatty acid is mostly found in olives and sesame [10, 11]. In an experimental study on the effect of high MUFA diet on cerebral ischemia, an improvement was observed in the neurological and motor function in acute ischemic stroke mice models receiving olive oil fatty acid supplements compared to placebo [3].

Targeting CBR2 has several effects on ROS-induced neuroinflammation, as it can reduce ROS/reactive nitrogen species (RNS) production in active glial cells, reduce vascular inflammation, improve blood-brain barrier (BBB) function, and inhibit leukocyte cell uptake and thus reduce nerve cell death [13].

The results of the present study confirmed the effect of OEA supplementations on the biomarkers of OS, inflammatory parameters (IL-6, CRP, and lipid profile), and renal and hepatic parameters in patients with acute ischemic stroke.

Our investigation indicated that OEA has remarkable effects on OS parameters, which can reduce OS and increase anti-OS marker in the patients with acute ischemic stroke. In a neuron-like SH-SY5Y cell line, Giusti et al. observed that 10 *μ*M oleocanthal, as a phenolic component of extra virgin olive oil (EVOO), neutralizes OS which was induced by H_2_O_2_, and leads to increased cell viability, decreased production of ROS, and an increased intracellular glutathione (GSH) level [27]. Furthermore, Tasset et al. demonstrated that EVOO, which represents 10% of calorie intake in the total standard daily diet of rats, reduces oxidative damage in Huntington's disease-like rat model induced by 3-nitropropionic acid (3NP) [28]. They further found that in all studied samples, 3NP increased lipid peroxides but decreased GSH levels [28]. However, their results revealed that EVOO reduced the level of LPO and blocked the GSH deficiency caused by 3NP in the striatum and other parts of the brain of Wistar rats [28]. In our study, TAC, TTG, and MDA levels had significant changes, confirming the antioxidant effects of OEA.

Previous studies have shown that mitochondrial CBR1 expression plays a unique role in cannabinoid-driven neuroprotection and might directly regulate mitochondrial ROS formation under this pathological condition [13]. Moreover, targeting CBR2 has several effects on ROS-induced neuroinflammation, as it can reduce ROS/RNS production in active glial cells, reduce vascular inflammation, improve blood-brain barrier function, inhibit leukocyte cell uptake, and consequently, decrease nerve cell death [13]. Therefore, the antioxidative effect of OEA in our study might be due to the activation of both CBR1 and CBR2.

Although the neuroprotective effects of OEA after acute cerebral ischemic injury in the experimental model have been reported, to the best of our knowledge, there are no clinical trials on humans to prove this concept [29]. It has been indicated that OEA (40 mg/kg, intraperitoneally (ip)) attenuates apoptosis by inhibiting the Toll-like receptor (TLR4)/NF-*κ*B and ERK1/2 signaling pathways in mice model of acute ischemic stroke [29]. N15, an analogue of OEA, has the ability to protect the brain against ischemic injury. Li et al. assessed both neuropreventive (50, 100, or 200 mg/kg, ip) and neurotherapeutic effects of N15 (200 mg/kg, ip) in mice model of acute ischemic stroke and also lipopolysaccharide- (LPS-) stimulated BV-2 microglial cells [30]. Furthermore, the anti-inflammatory properties of N15 may contribute to its neuroprotective effects on cerebral ischemia, at least in part, by boosting PPAR/dual signaling and suppressing the activation of the NF-*κ*B, STAT3, and ERK1/2 signaling pathways [30]. These data imply that OEA might be a promising therapy option for ischemic stroke prevention and treatment.

The evaluation of inflammatory parameters revealed that in the OEA 300 mg/day group, the IL-6 and CRP levels were significantly different before and after OEA administration. Contrarily, these levels were not significantly different in other groups before and after OEA administration. Between-group analysis showed that IL-6 in patients receiving OEA 300 mg/day was statistically lower than its level in patients who received placebo. A controlled clinical trial evaluated the anti-inflammatory and antioxidative effects of OEA (250 mg/day) on obesity, as well as assessing LPO, TAC, CRP, IL-6, and TNF-*α* levels. Based on the results, IL-6 and TNF-*α* concentrations were significantly reduced in the intervention group, but other changes were not significantly different [31]. Sayd et al. demonstrated that systemic administration of OEA (10 mg/kg, ip) could decrease levels of IL-1*β* and IL-6 as proinflammatory cytokines and also markers of nitrosative/OS nitrites and MDA which was induced by LPS (0.5 mg/kg, ip) in rats [32]. In another study, Xu et al. investigated the anti-inflammatory effect of different doses of OEA (10 *μ*M, 20 *μ*M, 50 *μ*M, and 100 *μ*M) and concluded that OEA reduces inflammatory cytokines (IL-6 and IL-8) and adhesion molecules on TNF-*α* (20 ng/ml)-induced inflammation in human umbilical vein endothelial cells through the activation of the CBR2 and PPAR-*α* [12]. Zhou et al. assessed both neuropreventive (10, 20,or 40 mg/kg, intragavage (ig)) and neurotherapeutic effects of OEA (40 mg/kg, ig) in a mice model of ischemic stroke and concluded that orally administered OEA protects mice from focal cerebral ischemic injury particularly BBB disruption by activating PPAR-*α* [33]. This impact is tremendously important since poststroke BBB disruption can exacerbate ischemic damage by raising edema and inducing bleeding [34]. During an acute ischemic stroke, cerebral edema is the most prevalent cause of neurological impairment and death [35]. The BBB's integrity can help avoid brain swelling and subsequent tissue damage. Since OEA is rapidly depleted in vivo due to hydrolysis, its therapeutic potential is limited. As a result, encapsulating OEA in a nanoparticulate structure like cubosomes, which may be utilized to target the BBB, protects it from hydrolysis and allows therapeutic amounts to reach the brain [36]. Another study by Wu et al. showed that the survival rate, behavioral score, cerebral infarct volume, edema degree, spatial learning, and memory capacity of stroke-model rats could all be improved greatly using endogenous OEA crystals loaded lipid nanoparticles [37]. Further studies should be done in order to find the most effective format for OEA administration.

Likewise, Luo et al. in both in vitro and in vivo experiments found that OEA (10-50 *μ*M) inhibits glial activation via modulating PPAR-*α* and promotes motor function recovery after brain ischemia [38]. Also, SUL (3 and 10 mg/kg) treatment, a stable OEA-modeled compound, in addition to PPAR-*α* antagonist, GW6471 (1 mg/kg), improves the brain damage and accompanying motor and cognitive impairments caused by hypoxia-ischemia in mice, most likely through regulating alterations in neuroinflammation/immune system mediators [39].

A meta-analysis demonstrated that acute kidney injury is a common complication following acute ischemic stroke and is associated with increased mortality following acute ischemic stroke [40]. Additionally, it was shown that impaired kidney function is associated with the presence of cerebral microbleeds in acute ischemic stroke [41], and renal dysfunction increases the risk of recurrent stroke in those patients [42]. Thus, kidney function preservation is crucially important in acute ischemic stroke patients. In our study, within-group analysis revealed that blood urea and creatinine, as the markers of renal function, had significantly changed in patients receiving OEA 300 mg/day, indicating that OEA might also be used as a renal protective factor in acute ischemic patients. Thus, OEA not only has no renal toxicity character, but also can prevent deleterious effects on kidneys.

In terms of the lipid profile, within-group analysis indicated a statistically significant decrease in the TG and cholesterol levels, while a rise in the HDL level in patients receiving OEA 300 mg/day. In terms of hepatic function, a statistically significant reduction was found in ALT in patients receiving OEA 300 mg/day. It is concluded that OEA has no hepatotoxic character, but can improve liver function at moderate doses (300 mg/day). In an experimental study on KDS-5104, a nonhydrolyzable lipid OEA analog, Thabuis et al. showed that the most significant bioindicator of OEA activity is adipose tissue fatty-acid translocase (FAT)/CD36 expression, which appears to be a determining player in the OEA fat-lowering response [43]. In addition, Fu et al. demonstrated PPAR-*α* and other PPAR-target genes, such as FAT/CD36, liver fatty-acid binding protein (L-FABP), and uncoupling protein-2 (UCP-2), are activated by subchronic OEA administration (5 mg/kg/day, ip, for two weeks) in Zucker rats [44]. Furthermore, OEA lowers hepatocyte neutral lipid content as well as blood cholesterol and TG levels. The findings imply that OEA might modulate lipid metabolism [44].

Similarly, Li et al. evaluated the effect of 17 weeks of OEA administration (5 mg/kg/day, ip) on nonalcoholic fatty liver disease (NAFLD) in Sprague Dawley rats and found that the treatment with OEA delayed the progression of NAFLD by regulating plasma TG and cholesterol levels and reducing ALT, AST, and inflammatory liver cytokines compared with controls [45]. On the other hand, the study of the liver and plasma tissue gene expression in these animal models showed that OEA increases lipid oxidation through PPAR-*α* activation [45]. The results of this study also represented that treatment with OEA inhibits the expression of genes involved in fat synthesis [45].

Higher levels of TG than HDL-C are associated with premature neuronal degradation, while lower ratios are linked with early clinical improvements. Several studies reported that TG/HDL-C can predict mortality and worsen clinical outcomes after acute ischemic stroke, and thus it is a simple and inexpensive indicator for predicting disease prognosis. The TG/HDL-C ratio is independently associated with mortality and poor prognosis in acute ischemic stroke patients [46, 47]. As such and considering the importance of lowering lipid profile, OEA might be considered in patients with acute ischemic stroke.

In terms of preclinical use of OEA in acute ischemic stroke treatment, an experimental study on Sprague Dawley rats demonstrated that chronic OEA therapy (30 mg/kg/day for 28 days) can promote neurogenesis in the hippocampus through increasing the expression of brain-derived neurotrophic factor (BDNF) and PPAR-*α* resulting in functional recovery of cognitive deficits and neuroprotective benefits against cerebral ischemic insult, indicating that OEA might be used therapeutically for cerebral ischemia [48].

In addition, by increasing collagen content and decreasing necrotic core size in plaques and also by modulating macrophage polarization both via the AMPK- PPAR-*α* pathway, OEA increased atherosclerotic plaque stability in both in vivo and in vitro experiments [49]. These data imply preventive role of OEA in ischemic events.

In general, a wide range of studies have pointed out the role of OS, inflammatory parameters, and lipid profile in the pathogenesis and prognosis of acute ischemic stroke. Accordingly, it is recommended that OEA, as a member of the NAE family, might be used to reduce the complications of acute ischemic stroke.

### 4.1. Limitations and Strengths

Despite the uniqueness of our findings, several limitations warn against extrapolating the findings too far including a relatively small number of patients enrolled in this study. More studies with larger sample sizes are warranted. The second drawback of this study is that each patient only had two samples obtained at baseline and 72 hours posttreatment. As a result, long-term understanding of the biomarker changes postacute ischemic stroke is not feasible. Third, the patients in our trial were given OEA supplements orally. As such, no conclusion can be withdrawn on intravenous (IV) OEA treatment effects. Fourth, testing the effects of OEA supplementation in a short length of time and lack of long-term follow-up is a disadvantage. Fifth, the effects of OEA on clinical outcome of these patients were not assessed. Sixth, the window for treatment with OEA in our trial was within 12 hours following the onset of stroke symptoms; earlier treatment may have different efficacy. Finally, IV thrombolytics are the only FDA-approved pharmacological treatment for acute ischemic stroke [50–60] and roughly half of our patients received this treatment given they were eligible. Thus, this treatment might have influenced our results.

Our study has the following advantages. It benefited from randomization, double-blinding, and the presence of a control group as a pilot clinical trial. Furthermore, the changes in inflammatory markers after treatment with OEA might be an indication of reduction in brain tissue inflammation happening after acute ischemic stroke.

## 5. Conclusion

Our results indicate that OEA add-on to standard acute ischemic stroke treatment improves the short-term inflammatory, OS status, and lipid and biochemical parameters in those patients, particularly in moderate dosage, 300 mg/day, which might lead to the better functional outcome. Our findings need to be confirmed in larger-scale studies with larger sample sizes and longer intervention duration and follow-up.

## Figures and Tables

**Table 1:** Demographic and clinical characteristics of the study participants.

		Number (%) or mean ± SD				
		All (*n* = 60)	Placebo (*n* = 20)	OEA 300 mg/day (*n* = 20)	OEA 600 mg/day (*n* = 20)	*P* value
Male/female		31/29	11/9	10/10	10/10	0.935

Age (years)		68.60 ± 2.10	65.4 ± 3.21	67.2 ± 2.65	69.7 ± 3.09	0.648

BMI (kg/m^2^)		26.9 ± 4.12	27.2 ± 3.71	26.4 ± 4.32	27.1 ± 3.45	0.762

Risk factors	Smoking	34 (56.6%)	11 (55%)	9 (45%)	14 (70%)	0.275
	Hypertension	51 (85%)	16 (80%)	17 (85%)	18 (90%)	0.676
	Hyperlipidemia	47 (78.3%)	14 (70%)	16 (80%)	15 (75%)	0.766
	CAD	35 (58.3%)	17 (85%)	12 (60%)	16 (80%)	0.155
	Diabetes mellitus	16 (26.6%)	5 (25%)	4 (20%)	7 (35%)	0.551
	Prior stroke	17 (28.3%)	7 (35%)	4 (20%)	6 (30%)	0.563
	DVT	6 (10%)	1 (5%)	2 (10%)	2 (10%)	0.804
	SBP, mmHg	149 ± 8.29	152 ± 11.01	149 ± 10.73	148 ± 12.14	0.742
	DBP, mmHg	87 ± 10.02	87 ± 8.73	89 ± 10.20	88 ± 12.65	0.933

Acute ischemic stroke event characteristics	NIHSS	5.2 ± 1.26	5.12 ± 1.72	5.18 ± 1.50	5.26 ± 1.37	0.790
	Infarct volume (cm^3^)	1.2 ± 0.87	1.1 ± 1.02	1.2 ± 0.98	1.4 ± 1.25	0.720
	mRS	2.1 ± 0.34	2.03 ± 0.53	2.12 ± 0.36	1.96 ± 0.27	0.502
	DTN, hours	8.3 ± 4.57	7.2 ± 5.19	7.9 ± 4.21	8.3 ± 4.71	0.191
	Thrombolytic therapy	27 (45%)	9 (45%)	8 (40%)	10 (50%)	0.817
	Nonthrombolytic therapy	33 (55%)	11 (55%)	12 (60%)	10 (50%)	0.817

Medications prior to admission	Antiplatelet therapy	18 (30%)	8 (40%)	4 (20%)	6 (30%)	0.386
	Anticoagulation therapy	18 (30%)	8 (40%)	4 (20%)	6 (30%)	0.386
	Statin therapy	47 (78.3%)	14 (70%)	16 (80%)	15 (75%)	0.766
	ACEI therapy	50 (83.3)	16 (80%)	17 (85%)	17 (85%)	0.877
	ARB therapy	4 (6.6%)	1 (5%)	2 (10%)	1 (5%)	0.765
	BB therapy	29 (48.3%)	11 (55%)	8 (40%)	10 (50%)	0.627
	CCB therapy	36 (60%)	12 (60%)	14 (70%)	10 (50%)	0.435
	Diuretics therapy	9 (15%)	3 (15%)	3 (15%)	3 (15%)	1.00
	Insulin with oral antidiabetic therapy	10 (16.6%)	3 (15%)	2 (10%)	5 (25%)	0.432
	Oral anti diabetic therapy	6 (10%)	2 (10%)	2 (10%)	2 (10%)	1.00

Underlying mechanism for stroke	Atherothrombotic	33 (55%)	12 (60%)	10 (50%)	11 (55%)	0.817
	Cardioembolic	9 (15%)	3 (15%)	2 (10%)	4 (20%)	0.676
	Lacunar infract	9 (15%)	3 (15%)	3 (15%)	3 (15%)	1.00
	Other causes	6 (10%)	2 (10%)	2 (10%)	2 (10%)	1.00
	Undetermined	3 (5%)	1 (5%)	1 (5%)	1 (5%)	1.00

Abbreviations: BMI, body mass index; CAD, coronary artery disease; DVT, deep vein thrombosis; SBP, systolic blood pressure; DBP, diastolic blood pressure; NIHSS, National Institutes of Health Stroke Scale; mRS, Modified Rankin scale; DTN, door-to-needle time; ACEI, angiotensin-converting-enzyme inhibitors; ARB, Angiotensin II receptor blockers; BB, beta blocker; CCB, calcium channel blocker.

**Table 2:** Laboratory characteristics of the study participants.

Lab parameter	Before or after receiving the capsule	Mean ± SD			*P* value
		Placebo (*n* = 20)	OEA 300 mg/day (*n* = 20)	OEA 600 mg/day (*n* = 20)	
Ur	Before	43.16 ± 10.98	52.25 ± 12.65	41.8 ± 12.63	0.796
	After	38.5 ± 12.34	42.25 ± 8.22	36.6 ± 14.04	
	*P* value	0.56	**0.046**	0.168	

Cr	Before	1.12 ± 0.32	1.34 ± 0.15	1.06 ± 0.17	0.466
	After	1.05 ± 0.23	1.17 ± 0.24	0.98 ± 0.13	
	*P* value	0.353	**0.030**	0.210	

TG	Before	109.5 ± 37.22	116.12 ± 44.87	108 ± 60.81	0.964
	After	77.75 ± 7.22	88.37 ± 27.72	74.5 ± 20.5	
	*P* value	0.126	**0.044**	0.449	

Chl	Before	149.5 ± 41.58	163.4 ± 37.62	156.5 ± 30.55	0.659
	After	135.25 ± 47.61	136.8 ± 28.60	140.9 ± 30.96	
	*P* value	0.217	**0.027**	0.138	

HDL	Before	47.85 ± 28.32	33.25 ± 6.38	35 ± 3.69	0.317
	After	47.42 ± 30.70	37.83 ± 5.89	39 ± 7.89	
	*P* value	0.834	**0.035**	0.288	

ALT	Before	16.71 ± 6.87	19.33 ± 4.50	15 ± 3.31	0.170
	After	18.57 ± 7.67	18.22 ± 4.65	14.2 ± 1.92	
	*P* value	0.374	**0.007**	0.374	

AST	Before	13.5 ± 2.51	19.66 ± 2.65	19.57 ± 7.16	0.424
	After	16 ± 8.28	19 ± 2.75	19.14 ± 6.98	
	*P* value	0.591	0.235	0.078	

IL-6	Before	2.42 ± 1.32	3.24 ± 1.81	3.20 ± 1.57	**0.035**
	After	3.18 ± 1.54	2.13 ± 0.59	3.26 ± 1.19	
	*P* value	0.216	**0.040**	0.888	

CRP	Before	4.25 ± 3.77	4.98 ± 3.16	4.17 ± 2.20	0.235
	After	4.59 ± 4.12	3.04 ± 1.54	4.44 ± 1.28	
	*P* value	0.831	**0.049**	0.743	

SOD	Before	0.24 ± 0.011	0.251 ± 0.013	0.25 ± 0.01	0.369
	After	0.25 ± 0.018	0.253 ± 0.023	0.24 ± 0.02	
	*P* value	0.187	0.792	0.435	

TAC	Before	0.59 ± 0.08	0.63 ± 0.082	0.70 ± 0.08	0.464
	After	0.61 ± 0.12	0.71 ± 0.099	0.73 ± 0.14	
	*P* value	0.637	**0.020**	0.478	

MDA	Before	0.089 ± 0.011	0.106 ± 0.021	0.097 ± 0.026	0.054
	After	0.090 ± 0.017	0.091 ± 0.018	0.080 ± 0.016	
	*P* value	0.820	**0.026**	**0.007**	

TTG	Before	0.140 ± 0.017	0.151 ± 0.016	0.155 ± 0.017	**0.022**
	After	0.134 ± 0.025	0.167 ± 0.016	0.151 ± 0.017	
	*P* value	0.494	**0.016**	0.266	

Abbreviations: Cr, Creatinine; Ur, Urea; Chl, Cholesterol; TG, Triglyceride; HDL, high-density lipoprotein; AST, aspartate transaminase; ALT, alanine transaminase; CRP, C-reactive protein; IL-6, interlukin-6; TAC, total antioxidant capacity; TTG, total thiol groups; MDA, malondialdehyde; SOD, superoxide dismutase.

**Table 3:** Frequency of drug-related adverse effects among patients in each groups.

	Number (%)				
	All (*n* = 60)	Placebo (*n* = 20)	OEA 300 mg/day (*n* = 20)	OEA 600 mg/day (*n* = 20)	*P* value
Nausea	16 (26.6%)	4 (20%)	6 (30%)	6 (30%)	0.711
Vomiting	11 (18.3%)	3 (15%)	4 (20%)	4 (20%)	0.895
Dyspepsia	24 (40%)	6 (30%)	8 (40%)	10 (50%)	0.435
Headache	26 (43.3%)	7 (35%)	10 (50%)	9 (45%)	0.622
Dizziness	15 (25%)	5 (25%)	5 (25%)	5 (25%)	1.000

## Data Availability

The authors confirm that the data supporting the findings of this study are available within the article.
